# Evaluation of Housing Affordability Among US Resident Physicians

**DOI:** 10.1001/jamanetworkopen.2023.20455

**Published:** 2023-06-27

**Authors:** Ryan C. L. Brewster, Alex Butler, Catherine D. Michelson, Jennifer Kesselheim

**Affiliations:** 1 Department of Pediatrics, Boston Children’s Hospital, Boston, Massachusetts; 2 Department of Pediatrics, Boston Medical Center, Boston, Massachusetts; 3 Department of Pediatric Oncology, Dana-Farber Cancer institute, Boston, Massachusetts

## Abstract

This cross-sectional study evaluates the extent of housing unaffordability among US residency programs.

## Introduction

Housing is the largest contributor to the rising cost of living and the regional variation therein in the US.^1^ Rental market fluctuations combined with graduate medical education (GME) payments for resident physician salaries and benefits that may not sufficiently support housing needs can worsen economic distress, contribute to burnout, and impact career decision-making.^2^ To help inform GME and institutional policy, we assessed the extent of housing unaffordability among US residency programs.

## Methods

We conducted a cross-sectional analysis of housing affordability and housing-related benefits for the 2022-2023 academic year. We obtained first postgraduate year (PGY-1) compensation information for all American College of Graduate Medical Education (ACGME)–accredited residency programs and sponsoring institutions from the American Medical Association FREIDA database (eAppendix in Supplement 1). We defined housing-related benefits as moving allowances and housing stipends. The Zillow Observed Rent Index (ZORI) was used to estimate June 2022 city-level rental prices, and rural-urban status was determined from the 2013 National Survey of Health Statistics Urban-Rural Classification Scheme for Counties. The study followed the STROBE reporting guideline and was exempted from Boston Children's Hospital institutional review board approval because all data were publicly available. Per the Common Rule, the requirement for informed consent does not apply because this was not human participant research.

A rent affordability index, calculated as the proportion of the local ZORI to the gross monthly PGY-1 salary, of 30% or greater is considered rent burdened by the US Department of Housing and Urban Development.^3^ We assessed trends in inflation-adjusted rental prices and PGY-1 salaries nationally from July 2000 to July 2022 with the Consumer Price Index for All Urban Consumers: Rent of Primary Residence in US City Average and the 2022 Association of American Medical Colleges Survey of Resident/Fellow Stipend and Benefits.

Institutional characteristics were examined for the association with housing affordability using multivariable logistic regression. We used annual growth rates and Mann-Kendall trend tests to evaluate changes in salary and rental prices over time. Statistical analyses were performed in R, version 3.5.2, with a significance threshold of 2-sided *P* < .05.

## Results

A total of 855 sponsoring institutions were included, most of which were community-based institutions with university affiliations (54.3%) supporting 1 to 2 ACGME-accredited residency programs (64.6%) (Table). A total of 511 sponsoring institutions (59.8%) were rent-burdened. Moving allowances and housing stipends were available at 246 (28.8%) and 117 (13.7%) institutions, respectively. Housing unaffordability was significantly associated with geographic region and urbanicity. Rent-burdened institutions were less likely to offer any housing-related benefits (adjusted odds ratio, 0.42; 95% CI, 0.28-0.61). Between 2000 and 2022, inflation-adjusted PGY-1 salaries decreased by 0.23% overall (annual growth rate, −0.01%; *P* = .002) (Figure), while there was a 17.8% increase (annual growth rate, 0.81%; *P* < .001) in inflation-adjusted rental prices.

**Table.** Characteristics of Sponsoring Institutions and Multivariable Logistic Regression of the Association Between Institution Characteristics and Housing Unaffordability

Characteristic	Institutions, No. (%)			Adjusted odds ratio (95% CI)
	Overall (N = 855)	Rent burdened^a^		
		Yes (n = 511)	No (n = 344)	
Region^b^				
East North Central	159 (18.6)	50 (9.8)	109 (31.7)	1.14 (0.48-2.84)
East South Central	33 (3.9)	13 (2.5)	20 (5.8)	5.02 (1.7-15.38)
Mid Atlantic	149 (17.4)	100 (19.6)	49 (14.2)	8.07 (3.48-20.23)
Mountain	60 (7.0)	43 (8.4)	17 (4.9)	16.75 (6.21-48.80)
New England	51 (6.0)	39 (7.6)	12 (3.5)	17.01 (6.17-50.98)
Pacific	116 (13.6)	109 (21.4)	7 (2.0)	92.51 (30.8-322.37)
South Atlantic	152 (17.8)	110 (21.6)	42 (12.2)	17.53 (7.42-45.05)
West North Central	55 (6.4)	10 (2.0)	45 (13.1)	1 [Reference]
West South Central	79 (9.3)	36 (7.1)	43 (12.5)	3.88 (1.56-10.28)
Rural-urban classification^c^				
Medium metropolitan	400 (46.8)	233 (45.7)	167 (48.5)	3.16 (2.01-5.03)
Rural	5 (0.6)	0	5 (1.5)	0 (0 to ∞)
Small metropolitan	184 (21.5)	56 (11.0)	128 (37.2)	1 [Reference]
Large metropolitan	258 (30.2)	215 (42.2)	43 (12.5)	16.25 (9.07-30.01)
Missing	7 (0.8)	6 (1.2)	1 (0.3)	NA
Institution type^d^				
Community based	168 (19.7)	87 (17.1)	81 (23.5)	1 [Reference]
Community based, university affiliated	464 (54.3)	277 (54.3)	187 (54.4)	1.25 (0.77-2.02)
Other	4 (0.5)	4 (0.8)	0	0 (0 to ∞)
University based	211 (24.7)	136 (26.7)	75 (21.8)	1.29 (0.68-2.46)
Missing	7 (0.8)	6 (1.2)	1 (0.3)	NA
Programs per institution, No.				
1-2	552 (64.6)	316 (62.0)	236 (68.6)	1 [Reference]
3-4	102 (11.9)	67 (13.1)	35 (10.2)	1.39 (0.79-2.49)
≥5	200 (23.4)	127 (24.9)	73 (21.2)	1.00 (0.59-1.67)
Offers any housing benefit^e^	307 (35.9)	151 (29.6)	156 (45.3)	0.42 (0.28-0.61)

Abbreviation: NA, not applicable.

^a^ As established by the Department of Urban and Housing Development, an institution was considered rent-burdened if the local monthly rental estimate (given by Zillow) constituted 30% or more of gross monthly postgraduate year 1 salary.

^b^ Geographic regions are defined by the US Census Bureau.

^c^ The National Center for Health Statistics Rural-Urban Classification Scheme assigns counties to different categories based on population size per the 2013 US census: large metropolitan (>1 million), medium metropolitan (250 000-999 999), small metropolitan (50 000-249 999), and rural (<50 000).

^d^ The American Medical Association FREIDA database classifies sponsoring institutions into different hospital types. Community-based programs do not take place in a university academic medical center or hospital with a medical school affiliation. Community-based, university-affiliated programs take place in a community hospital that is affiliated with an academic medical center but is not a primary affiliate or is geographically separate from the academic medical center. University-based programs take place in a hospital that serves as a primary affiliate of the medical school.

^e^ Housing-related benefits encompassed moving allowances (246 [28.8%] overall) and housing stipends (117 [13.7%] overall).

**Figure.**
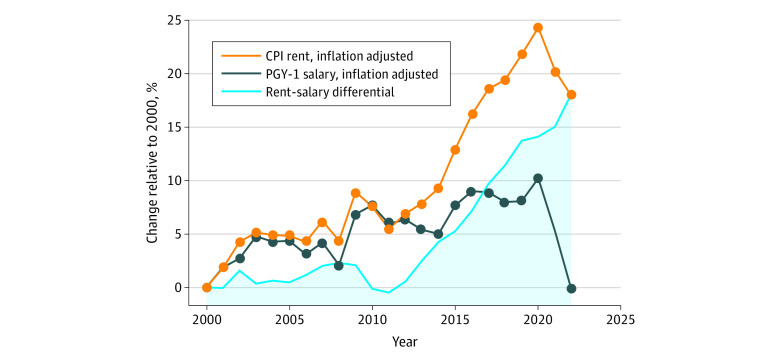
Percent Growth of Consumer Price Index (CPI) Rent and Postgraduate Year 1 (PGY-1) Salary From 2000 to 2022 Data were indexed to the year 2000. Values were adjusted to inflation based on the CPI for All Urban Consumers. The rent-salary differential represents the absolute difference between CPI rent and PGY-1 salary.

## Discussion

This study found that residents faced a widening gap in housing affordability with marked geographic variation, consistent with studies showing that cost of living indices poorly correlate with trainee salaries.^4,5^ Moreover, the growth in rental prices outpaced that of resident earnings. Yet, housing-related benefits were scarcely available and even less so at rent-burdened institutions.

Rising housing prices have affected many sectors; however, education debt, training requirements, long working hours, and general lack of control over compensation may make residents uniquely vulnerable to financial challenges.^6^ Our findings suggest that there remain opportunities for GME reimbursements and sponsoring institutions to adjust for cost-of-living considerations and provide supplemental financial benefits, particularly in urban areas. Of note, resident labor union participation has been shown to be associated with improved access to housing stipends.^6^

A limitation is that the FREIDA database does not have universal capture of all residency programs; also, because the magnitude of benefits is not listed, it is difficult to assess their financial impact. The lack of resident-level housing data, such as housing type and living arrangements, highlights a future research direction. Ultimately, institutional- and national-level policy advocacy appears to be needed to ensure equitable compensation that reflects local economic conditions.

## Notes

Supplement 1.
eMethods

eReferences

Supplement 2.
Data Sharing Statement
