# Supplementary material for: Evaluation of Housing Affordability Among US Resident Physicians
Source: JAMA Netw Open. 2023 Jun 27;6(6):e2320455. doi: 10.1001/jamanetworkopen.2023.20455 (PMC10300675; doi:10.1001/jamanetworkopen.2023.20455)
Supplement: Supplement 1. — eMethods eReferences [file jamanetwopen-e2320455-s001.pdf]

Supplemental Online Content

Brewster RCL, Butler A, Michelson CD, Kesselheim J. Evaluation of housing affordability among US resident physicians. *JAMA Netw Open.* 2023;6(6):e2320455. doi:10.1001/jamanetworkopen.2023.20455

eMethods

eReferences

This supplemental material has been provided by the authors to give readers additional information about their work.

© 2023 Brewster RCL et al. *JAMA Network Open.*

eMethods.

<u>Zillow Observed Rent Index</u>

The Zillow Observed Rent Index (ZORI) is calculated with a single-unit, repeat transaction analysis and measures changes in rental appreciation over time. Additional weights from the American Community Survey (ACS) are assigned to control for selection biases, namely the overrepresentation of expensive units on private listing sources. [1] The ZORI for a given region is dollar denominated by determining the mean of the middle quintile of listed rents for all homes and apartments. A full description of Zillow's methodology is detailed on their website.

<u>Reconciling Residency Program and Sponsoring institution Characteristics</u>

In this analysis, the term *residency program* is defined as an accredited medical residency program at an approved, qualifying institution of which some portion of the education occurs as direct instruction. The term *sponsoring institution* is defined as an entity that oversees, supports, and administers one or more ACGME-accredited residency/fellowship programs.

Resident salaries and benefits are standardized within a sponsoring institution, regardless of specialty, as stipulated by direct graduate medical education (GME) payments. There were discrepancies in resident salaries across specialties in 4.1% of institutions reporting to FREIDA. To reconcile these differences, we selected the highest salary reported by a sponsoring institution and applied that value to all programs within that institution. A total of 5.1% of institutions disclosed inconsistent housing-related benefits. If at least one specialty provided a particular benefit, it was assumed that all specialties would receive the same benefit. We manually corroborated salary and benefit packages with program website and recruitment materials, where available.

<u>Consumer Price Index</u>

Consumer Price Index (CPI), as defined by the Bureau of Labor and Statistics, measures the monthly change in prices paid by U.S. consumers. [2] This number is a weighted average of prices for a collection of goods and services representative of aggregate U.S. consumer spending. Two indices are published each month: the CPI for All Urban Consumers (CPI-U), which represents 93% of the U.S. population not living in remote rural areas, and the CPI for Urban Wage Earners and Clerical Workers (CPI-W), which covers 29% of the U.S. population living in households with income derived predominantly from clerical employment or jobs with an hourly wage.

Within the overall CPI, one of the largest contributors is the index for shelter, or the service that a housing unit provides its occupants. Shelter is tracked via two measurements within the CPI: owners' equivalent rent (OER) and rent of primary residence. OER represents the amount of rent that would have to be paid in order to

© 2023 Brewster RCL et al. *JAMA Network Open.*

substitute a currently owned house as a rental property, in effect the amount of monthly rent that would be equivalent to the monthly expense of a property. Rent of primary residence represents changes in rent paid for renters of their current shelter.

To best represent the change in housing most commonly utilized by resident physicians, the CPI-U: Rent of Primary Residence was selected as the majority of residents reside in urban areas and rent their shelter as compared to owning it (e.g., house or condominium).

eReferences

1. Zillow. *Methodology: Zillow Observed Rent Index (ZORI)*.; 2022. https://www.zillow.com/research/methodology-zori-repeat-rent-27092/

2. U.S. Bureau of Labor Statistics. *Consumer Price Index*. https://www.bls.gov/cpi/#:~:text=January%202023%20CPI%20weight%20update&text=The%20Consumer%20Price%20Index%20(CPI,U.S.%20and%20various%20geographic%20areas.

3. National Center for Health Statistics. *Https://Www.Cdc.Gov/Nchs/Data_access/Urban_rural.Htm*. https://www.cdc.gov/nchs/data_access/urban_rural.htm

© 2023 Brewster RCL et al. *JAMA Network Open.*
