# Supplementary material for: Evaluation of Housing Affordability Among US Resident Physicians
Source: JAMA Netw Open. 2023 Jun 27;6(6):e2320455. doi: 10.1001/jamanetworkopen.2023.20455 (PMC10300675; doi:10.1001/jamanetworkopen.2023.20455)
Supplement: Supplement 2. — Data Sharing Statement [file jamanetwopen-e2320455-s002.pdf]

Data Sharing Statement

Brewster. Evaluation of Housing Affordability Among US Resident Physicians. *JAMA Netw Open*. Published June 27, 2023. doi:10.1001/jamanetworkopen.2023.20455

Data
Data available: No
